# Misdiagnosis of popliteal leiomyosarcoma: A case report and literature review

**DOI:** 10.1097/MD.0000000000042519

**Published:** 2025-05-16

**Authors:** Lan Zhang, Shiwei Liu, Hongyu Wang, Yimin Zhang

**Affiliations:** a School of Clinical Medicine, Shandong Second Medical University, Weifang, Shandong, China; b Department of Joint surgery, The First Affiliated Hospital of Shandong Second Medical University, Weifang, Shandong, China.

**Keywords:** leiomyosarcoma, malignant tumors, popliteal fossa, tumor resection

## Abstract

**Rationale:**

Leiomyosarcoma (LMS) is usually derived from the uterus, retroperitoneum, or intra-abdominal region. However, it is relatively rare in the popliteal fossa and can easily lead to misdiagnoses in clinical practice.

**Patient concerns:**

A 52-year-old female was admitted to the hospital with a “left popliteal fossa mass that had persisted for 8 days.”

**Diagnoses:**

The patient was diagnosed with benign popliteal fossa tumor following admission. The final pathological diagnosis was a malignant popliteal LMS.

**Interventions:**

The patient underwent 2 operations. First, the tumor was completely removed with an integral envelope and sent for pathological examination; the diagnosis was LMS. Subsequently, a 2nd extended resection is performed.

**Outcomes:**

Postoperatively, the patient received relevant medical antitumor therapy and was followed-up for 3 months, with no recurrence or metastasis.

**Lessons:**

Cases of LMS can develop in uncommon sites, such as the popliteal fossa, and present in the extremities, although rarely. Preoperative needle biopsy is recommended to confirm the nature of this type of tumor and ensure the range of excision.

## 1. Introduction

Leiomyosarcoma (LMS) accounts for 0.7% of all malignancies and 5% to 10% of soft tissue sarcomas.^[1]^ While 75% to 80% of cases arise in the uterus or retroperitoneum,^[2]^ soft tissue LMS in the lower extremities is exceedingly rare, with an estimated incidence of <1% among extremity sarcomas. Only 12 cases of popliteal fossa LMS have been reported in the past decade, highlighting the diagnostic challenges in such atypical locations. An extensive and comprehensive literature search revealed only a few cases of LMS in the soft tissues of the lower extremities.^[3,4]^ Due to its low incidence in the lower extremities, it often leads to misdiagnosis in clinical practice, which affects the therapeutic outcome.

Here, we report the case of a 52-year-old female who presented with a soft tissue mass in the left popliteal fossa. As the initial diagnosis was benign, the primary surgery was resection of the tumor with its integral envelope. However, postoperative pathological examination confirmed that the real nature of this “benign” tumor was LMS. Therefore, a 2nd extended resection was performed and radiotherapy was initiated. A follow-up study performed over 3 months showed no recurrence or metastasis.

## 2. Case presentation

**Figure 1.**
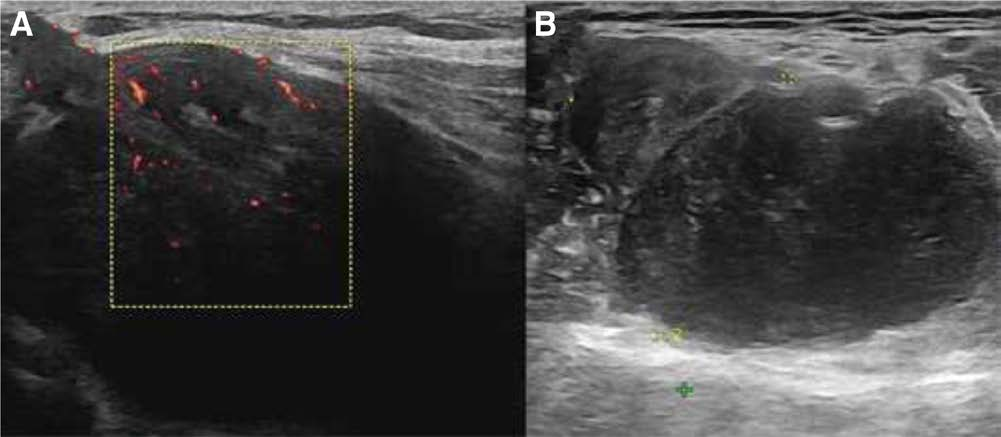
Local scanning of the left popliteal fossa revealed a 4.9 × 3.2 cm hypoecho in the deep surface of the popliteal artery in the peroneal muscle layer, with a clear boundary, regular shape, and visible blood flow signals.

**Figure 2.**
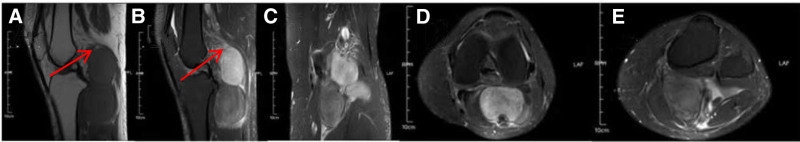
Sagittal T1WI (A) and fat-suppressed T2WI (B) showing 2 well-circumscribed masses in the popliteal fossa (arrows). Coronal (C) and axial (D, E) sequences confirm no adjacent tissue invasion.

A 52-year-old female patient was admitted to the hospital with an “8-day” history of a left popliteal fossa mass. Eight days previously, a mass was inadvertently found in the left popliteal fossa. She visited our hospital for a consultation. Physical examination revealed an egg-sized mass in the posterior popliteal fossa of the left knee with soft texture, clear boundary, mobility, no obvious tenderness around or radiating pain along the lower limb, and no palpable inguinal lymph node enlargement. Muscle strength and tension of the affected limbs were normal. The laboratory test results revealed no abnormalities. Local scanning of the left popliteal fossa revealed a 4.9 × 3.2 cm hypoechoic area in the deep surface of the popliteal artery among the peroneal muscle layer, with a well-defined boundary, regular shape, and visible blood flow signals (Fig. 1). Plain magnetic resonance imaging (MRI) revealed abnormal signal lesions in the left popliteal fossa (Fig. 2). No other lesions were noted.

**Figure 3.**
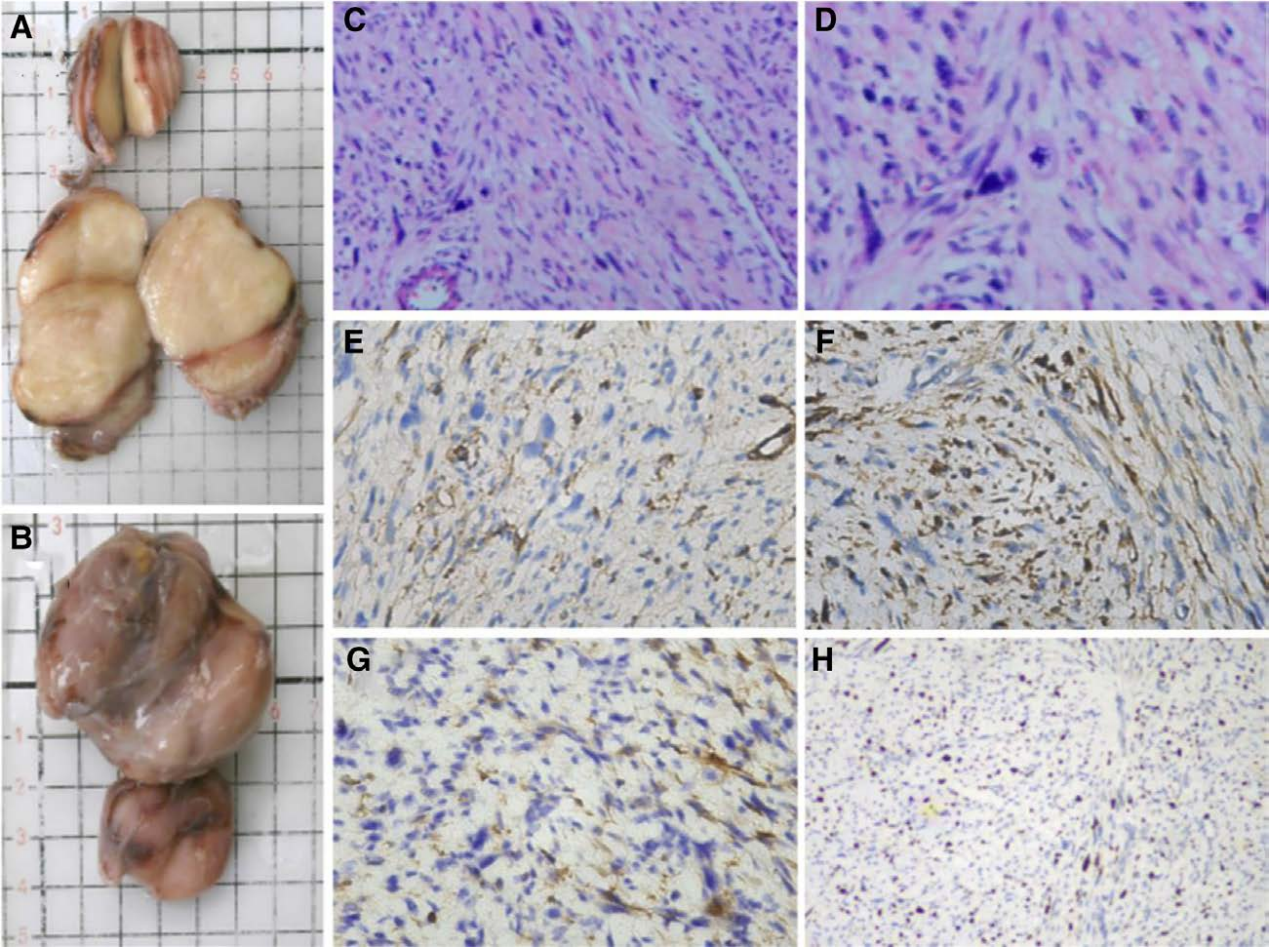
Macroscopic and microscopic view of the tumor. The tumor is a grayish-brown irregular soft tissue (A), and the medial section of the tumor (B). HE staining (×200): Microscopically with typical “cigar-shaped” nuclei and eosinophilic cytoplasm (C, D). Immunohistochemistry, original magnification (×200): The tumor was partially positive for SMA (E), positive for desmin (F), partially positive for calponin (G), and Ki-67 proliferative index of 20% (H). SMA = smooth muscle actin.

The patient underwent excision of the tumor following preoperative preparation. During the maneuver, a 15 cm arc-shaped incision was made in the left popliteal fossa, and 2 isolated gray-brown tumor bodies were located in the posterolateral pant of the popliteal fossa, both with a complete envelope and without adhesion to the surrounding tissues, including vessels or nerves. The larger 1 was 5 × 4 × 3 cm, and the other 1 × 1 × 1 cm. The 2 occupying lesions were completely removed and sent for pathological examination. Histopathological analysis revealed intersecting fascicles of spindle cells with marked nuclear atypia, eosinophilic cytoplasm, and cigar-shaped nuclei (Fig. 3C, D). Focal necrosis and a mitotic rate of 12 per 10 high-power fields were observed. Immunohistochemistry demonstrated diffuse strong positivity for smooth muscle actin (SMA; Fig. 3E) and desmin (Fig. 3F), focal positivity for calponin (Fig. 3G), with Ki-67 proliferative index of 20% (Fig. 3H).

Differential diagnoses included benign leiomyoma, schwannoma, and myxoid liposarcoma. Leiomyoma was excluded due to nuclear atypia and high mitotic activity. Schwannoma typically expresses S100 (negative here), while myxoid liposarcoma lacks SMA/desmin expression and may show MDM2 amplification.^[5]^

The patient underwent a 2nd enlarged excision and radiotherapy was administered, followed by wound healing for complex treatment. The follow-up period was just 3 months, and the patient had a good recovered well.

## 3. Discussion

LMS is a rare soft tissue sarcoma originating from smooth muscle cells, accounting for approximately 0.7% of all malignant tumors and 5% to 10% of soft tissue sarcomas. It can be classified into 3 subtypes according to their location: soft tissue LMS, cutaneous LMS, and vascular LMS. Although LMS is generally present in the retroperitoneum, abdominal cavity, and mediastinum.^[6]^ A small number of cases occur in other sites, among which the lower limbs are the most common predilection of this subtype.^[7]^ Cases involving the femoral vein^[8]^ and popliteal artery^[9]^ have been reported by Zhang et al and Voulalas et al, respectively. De Biasi^[3]^ and Gao^[4]^ also reported soft tissue LMS in the thighs. However, LMSs of >5 cm in the popliteal fossa have rarely been reported.

This paper presents a case of LMS in the subcutaneous soft tissue of the left popliteal fossa that was inadvertently discovered without a history of substantial pain and edema^[8,9]^ or a history of intermittent claudication of the lower extremities.^[9]^ Following admission, the patient was primarily diagnosed with a benign tumor before and during the 1st operation. However, a subsequent pathological examination confirmed the malignant nature of the tumor, and a 2nd extended resection was performed.

In retrospect, we have the ability to make an initial diagnosis of the tumor using basic imaging techniques, such as ultrasound, computed tomography (CT) tomography, and MRI. For example, MRI is a suitable choice to identify tumor features and determine their size, extension, and anatomical relationships^[10]^ and is also very useful for preoperative planning. However, this type of check is not helpful for accurately distinguishing benign from malignant lesions. Positron emission tomography/CT may be helpful for staging, prognosis, grading, and determining response to neoadjuvant therapy.^[11]^ Imaging features of popliteal LMS include well-circumscribed masses with heterogeneous T2 signal and peripheral enhancement on contrast MRI. In this case, the lesions showed long T1 and T2 signals without infiltration (Fig. 2), mimicking benign tumors. Positron emission tomography/CT may aid in detecting metabolic activity suggestive of malignancy.^[11]^

The definitive diagnosis of LMS is based on histopathological findings. They are characterized by pleomorphic hyperchromatic spindle cells with typical “cigar-shaped” nuclei and eosinophilic cytoplasm.^[11]^ Immunohistochemical studies are helpful for establishing these features. According to WHO 2020 criteria, LMS diagnosis requires: morphological evidence of smooth muscle differentiation; immunohistochemical positivity for ≥2 smooth muscle markers (SMA, desmin, calponin); exclusion of mimics (e.g., gastrointestinal stromal tumor, malignant peripheral nerve sheath tumor).^[5]^

Prognostic factors for LMS include tumor size (>5 cm), mitotic count (>10/10 high-power fields), and margin status.^[5]^ Here, the tumor measured 5 cm but achieved R0 resection after reoperation, correlating with a 5-year survival rate of 60% to 70%.^[12]^ Recent studies demonstrate that adjuvant radiotherapy improves local control rates in extremity LMS.^[13]^ While surgical resection remains the cornerstone, neoadjuvant chemotherapy (e.g., gemcitabine-docetaxel regimen) may be considered for large tumors (>10 cm) to facilitate margin-negative resection.^[14]^ Adjuvant radiotherapy (60 Gy) was administered for local control,^[15]^ while chemotherapy (doxorubicin) was reserved for metastatic disease.^[16]^

## 4. Conclusions

Preoperative core needle biopsy is mandatory for popliteal fossa masses, even with benign imaging features. Multidisciplinary collaboration (radiology, pathology, oncology) optimizes diagnostic accuracy and treatment planning. Long-term follow-up (>5 years) is recommended to monitor late recurrences.^[17]^

## Author contributions

**Resources:** Shiwei Liu, Hongyu Wang.

**Writing – review & editing:** Yimin Zhang.

**Writing – original draft:** Lan Zhang.

## References

[1] Rizwan T Ahmed J Shaikh FH Malik F Ullah S. Giant leiomyosarcoma arising in posterior thigh: management of a rare case. Cureus. 2020;12:e10146. 33014644 10.7759/cureus.10146 PMC7526762

[2] Arnold LM 3rd Burman SD O-Yurvati AH. Diagnosis and management of primary pulmonary leiomyosarcoma. J Am Osteopath Assoc. 2010;110:244–6. 20430913

[3] De Biasi G Cazzato G Colagrande A Maiorano E Ingravallo G. Giant soft tissue leiomyosarcoma of the left lower extremity: case presentation with a review of the literature. Cureus. 2023;15:e37058. 37153250 10.7759/cureus.37058 PMC10155594

[4] Chuanping G Weiwei F. Recurrent, giant subcutaneous leiomyosarcoma of the thigh. Radiol Case Rep. 2015;10:18–21. 26649111 10.1016/j.radcr.2015.06.005 PMC4633980

[5] WHO Classification of Tumors Editorial Board. Female Genital Tumors. 5th ed. IARC; 2020:247.

[6] Marko J Wolfman DJ. Retroperitoneal leiomyosarcoma from the radiologic pathology archives. Radiographics. 2018;38:1403–20. 30207936 10.1148/rg.2018180006 PMC6166742

[7] Lee MS Shi CR Sauer M Laga AC Talia J Nambudiri VE. Bilateral lower extremity induration in a patient with leiomyosarcoma. Lancet Oncol. 2021;22:e466. 34592196 10.1016/S1470-2045(21)00469-1

[8] Zhang M Yan F Huang B Wu Z Wen X. Multimodal ultrasonographic assessment of leiomyosarcoma of the femoral vein in a patient misdiagnosed as having deep vein thrombosis: a case report. Medicine (Baltimore). 2017;96:e8581. 29145269 10.1097/MD.0000000000008581 PMC5704814

[9] Voulalas G Giannakakis S Maltezos C. Acute ischemia of extremity as a first manifestation of peripheral artery leiomyosarcoma: report of a case and review of the literature. Ann Vasc Surg. 2016;34:268.e9–12. 10.1016/j.avsg.2015.11.027 26923155

[10] Killoran TP Wells WA Barth RJ Goodwin DW. Leiomyosarcoma of the popliteal vein. Skeletal Radiol. 2003;32:174–8. 12605285 10.1007/s00256-002-0498-8

[11] Georgeanu VA Pletosu RI Vlădescu TC Bondari S Crăciunescu A Russu OM. Primary bone leiomyosarcoma of distal femur: case report and literature review. Rom J Morphol Embryol. 2022;63:569–74. 36588496 10.47162/RJME.63.3.12 PMC9926145

[12] Gronchi A Miah AB Dei Tos AP. Soft tissue sarcomas: ESMO-EURACAN clinical practice guidelines. Ann Oncol. 2021;32:1348–65. 34303806 10.1016/j.annonc.2021.07.006

[13] Gronchi A Strauss DC Miceli R. Variability in patterns of recurrence after resection of primary retroperitoneal sarcoma (RPS): a report on 1007 patients from the multi-institutional collaborative RPS working group. Ann Surg. 2016;263:1002–9. 26727100 10.1097/SLA.0000000000001447

[14] George S Merriam P Maki RG. Multicenter phase II trial of gemcitabine and docetaxel in patients with metastatic soft tissue sarcomas: results of sarcoma alliance for research through collaboration study 002 [corrected]. J Clin Oncol. 2006;24:3044–50. 10.1200/JCO.2006.10.4117 17602081

[15] Poosiripinyo T Sukpanichyingyong S Salang K Sumananont C Chobpenthai T. Recurrence leiomyosarcoma of the popliteal vein: a rare soft tissue sarcoma. Case Rep Surg. 2023;2023:2788584. 36845634 10.1155/2023/2788584 PMC9949943

[16] Divyambika CV Sathasivasubramanian S Krithika CL Malathi N Prathiba D. Pediatric oral leiomyosarcoma: rare case report. J Cancer Res Ther. 2012;8:282–5. 22842376 10.4103/0973-1482.98990

[17] Clark MA. Long-term follow-up of soft tissue sarcoma. J Surg Oncol. 2018;117:33–40.29315649

